# Navigating Nursing Home Workforce Challenges: Leaders’ Voices for Sustaining Person-Centered Care

**DOI:** 10.1093/geroni/igaf122.010

**Published:** 2025-12-31

**Authors:** Nahida Akter, Salma Akter, Lucy Mondol, Asifa Khatun, Liza Behrens, Kimberly Van Haitsma

**Affiliations:** The Pennsylvania State University, University Park, Pennsylvania, United States; Wayne State University, Detroit, Michigan, United States; Grameen Caledonian College of Nursing, Dhaka, Dhaka, Bangladesh; Dhaka Nursing College, Dhaka, Dhaka, Bangladesh; The Pennsylvania State University, University Park, Pennsylvania, United States; The Pennsylvania State University, University Park, Pennsylvania, United States

## Abstract

Nursing home (NH) leaders face ongoing challenges in addressing workforce shortages while ensuring federally mandated person-centered care (PCC) for residents. This study explores NH leaders’ voices in managing staffing challenges while attempting to honor residents’ daily care preferences-a key aspect of PCC. This qualitative descriptive study utilized semi-structured interviews (n = 14) from four NHs in Pennsylvania. NH leaders (administrators, clinical directors and supervisors) participated in in-depth individual interviews, which were then evaluated using inductive thematic coding structured according to the Nurse Staffing Framework. Our data revealed four key themes. First, confirming honoring residents’ preferences is essential for PCC, with leaders emphasizing its benefits for resident well-being and staff satisfaction despite challenges such as perceived family interference and resource limitations. Second, workforce shortages pose a significant barrier to PCC, driven by negative perceptions of nursing, recruitment difficulties, and state-mandated staffing ratios, leading to staff overload and increased reliance on agency staff. Third, leaders employ various workforce management strategies, including pay adjustments, flexible scheduling, and staff recognition, to improve staff retention and maintain care quality. Finally, advocacy for policy reforms is perceived as crucial, with leaders stressing the need for regulatory changes to enhance staffing levels, workforce stability, and the overall sustainability of high-quality, resident-centered care in NHs. The paper will discuss insights into how workforce shortages impinge upon PCC, suggest effective workforce management strategies, as well as policy and regulatory reforms to strengthen both workforce stability and ensure the consistent delivery of PCC to residents in NHs.

